# The transcription factors *ADR1* or *CAT8* are required for RTG pathway activation and evasion from yeast acetic acid-induced programmed cell death in raffinose

**DOI:** 10.15698/mic2016.12.549

**Published:** 2016-12-02

**Authors:** Luna Laera, Nicoletta Guaragnella, Maša Ždralević, Domenico Marzulli, Zhengchang Liu, Sergio Giannattasio

**Affiliations:** 1 National Research Council of Italy, Institute of Biomembranes and Bioenergetics, Bari, Italy.; 2 Department of Biological Sciences, University of New Orleans, New Orleans, LA, USA.

**Keywords:** yeast, programmed cell death, mitochondrial retrograde pathway, glucose, carbon catabolite repression, acetic acid

## Abstract

Yeast *Saccharomyces cerevisiae* grown on glucose undergoes programmed cell death (PCD) induced by acetic acid (AA-PCD), but evades PCD when grown in raffinose. This is due to concomitant relief of carbon catabolite repression (CCR) and activation of mitochondrial retrograde signaling, a mitochondria-to-nucleus communication pathway causing up-regulation of various nuclear target genes, such as *CIT2*, encoding peroxisomal citrate synthase, dependent on the positive regulator *RTG2* in response to mitochondrial dysfunction. CCR down-regulates genes mainly involved in mitochondrial respiratory metabolism. In this work, we investigated the relationships between the RTG and CCR pathways in the modulation of AA-PCD sensitivity under glucose repression or de-repression conditions. Yeast single and double mutants lacking *RTG2* and/or certain factors regulating carbon source utilization, including *MIG1*, *HXK2*, *ADR1*, *CAT8*, and *HAP4*, have been analyzed for their survival and *CIT2* expression after acetic acid treatment. *ADR1 *and *CAT8* were identified as positive regulators of *RTG*-dependent gene transcription. *ADR1* and *CAT8* interact with *RTG2* and with each other in inducing cell resistance to AA-PCD in raffinose and controlling the nature of cell death. In the absence of *ADR1* and *CAT8*, AA-PCD evasion is acquired through activation of an alternative factor/pathway repressed by *RTG2,* suggesting that* RTG2* may play a function in promoting necrotic cell death in repressing conditions when RTG pathway is inactive. Moreover, our data show that simultaneous mitochondrial retrograde pathway activation and *SNF1*-dependent relief of CCR have a key role in central carbon metabolism reprogramming which modulates the yeast acetic acid-stress response.

## INTRODUCTION

Glucose is by far the preferred carbon source of the budding yeast *Saccharomyces cerevisiae*, because glucose metabolic regulation dictates the organism's distinctive fermentative lifestyle—aerobic fermentation (the Crabtree effect) 1,2*.* The phenomenon of glucose repression is a global regulatory mechanism causing inhibition of transcription of a large set of genes mainly involved in mitochondrial respiratory metabolism 3,4,5,6, which is known as the carbon catabolite repression (CCR) pathway. CCR is mediated, in part, by the crosstalk between two glucose signaling pathways: the *RGT2*/*SNF3* axis responsible for glucose uptake 7,8,9; and the *SNF1*/*MIG1* axis that negatively regulates the genes involved in respiratory metabolism and the use of alternative sugars 3,10,11. Since energy generation by fermentation is inefficient in terms of the ATP yield, yeast cells pump a large amount of glucose through glycolysis by enhancing its uptake, the first, rate-limiting step of glucose metabolism 12. The CCR pathway interacts with other intracellular signaling pathways inducing a signaling network which senses the constantly fluctuating nutrient content of the environment, determining cell growth, stress resistance and metabolism 13.

**Figure 1:**
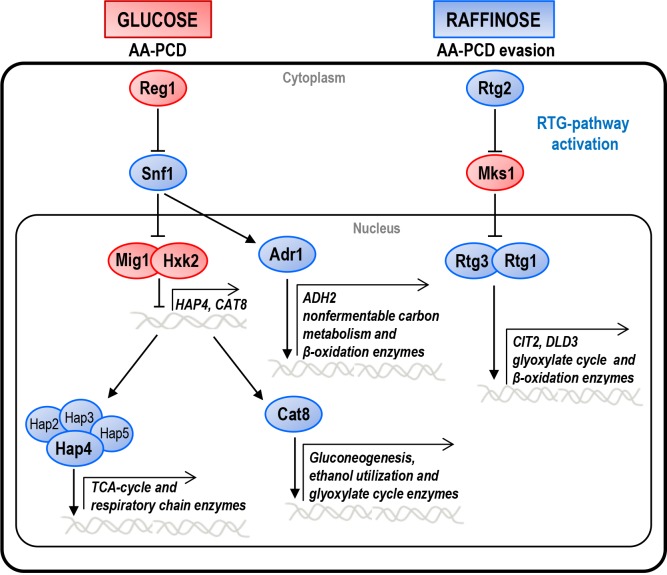
FIGURE 1: CCR and RTG pathway network. Schematic overview of the CCR and RTG pathways in yeast cells growing either in glucose, in which cells undergo AA-PCD, or in raffinose, in which cells evade AA-PCD. For details see text. The factors regulating either pathway are indicated. Blue and red shapes indicate proteins active in raffinose or in glucose, respectively. Major genes whose transcription is controlled by each pathway are indicated.

Yeast cells grown in glucose (GLU-WT) undergo acetic acid-induced programmed cell death (AA-PCD) sharing many morphological and biochemical features with mammalian apoptosis, including DNA fragmentation, phosphatidylserine (PS) externalization and mitochondrial dysfunction (for ref see 14,15). We have demonstrated that energy metabolism influences AA-PCD. Indeed, yeast cells grown in raffinose (RAF-WT) evade AA-PCD due to concomitant relief from CCR and activation of mitochondrial retrograde (RTG) signaling 16, a mitochondria-to-nucleus communication pathway causing up-regulation of a broad array of nuclear target genes in response to mitochondrial dysfunction dependent on *RTG2 *and* MKS1*, coding for positive and negative regulator of the pathway 17 (Fig. 1). The RTG pathway has been implicated in the intracellular signaling network linking mitochondrial function and cellular metabolism to several physiological processes such as ageing 18, PCD 16, autophagy 19 and ceramide metabolism 20.

There are two groups of RTG-target genes. The first group includes *CIT2*, encoding peroxisomal citrate synthase, and *DLD3*, encoding D-lactate dehydrogenase, whose transcription is strictly dependent on the heterodimeric transcription factor Rtg1/3 and shows a robust retrograde response 21. The second group includes a number of tricarboxylic acid (TCA) cycle genes, which do not show an obvious retrograde response, their transcriptional control switching from the Hap2/3/4/5 complex to Rtg1/3 in response to a reduction or loss of respiratory function 22. Hap2/3/4/5 complex is a transcription factor that is subject to glucose repression together with Adr1 and Cat8 23,24 (Fig. 1). Hap4 regulates the expression of genes involved in mitochondrial respiratory metabolism including those encoding the TCA cycle enzymes and components of the respiratory chain complex genes 25,26,27. Adr1 regulates genes involved in peroxisomal biogenesis, β-oxidation and utilization of non-fermentable carbon sources 28, whereas Cat8 regulates genes encoding gluconeogenic and glyoxylate cycle enzymes 29.

In this work, we used yeast to gain further insights into the relationships between the RTG and CCR pathways in the modulation of AA-PCD sensitivity under glucose repression or de-repression conditions, i.e. in glucose-grown (GLU-WT) cells, which undergo AA-PCD, or in raffinose-grown (RAF-WT) cells, which evade AA-PCD. To this aim we used yeast mutants lacking the RTG-signaling regulators *RTG2*, *MKS1* and/or certain transcription factors regulating carbon source utilization.

## RESULTS

### Genetic inactivation of CCR by *MIG1* or *HXK2* deletion does not affect sensitivity of yeast to AA-PCD

**Figure 2:**
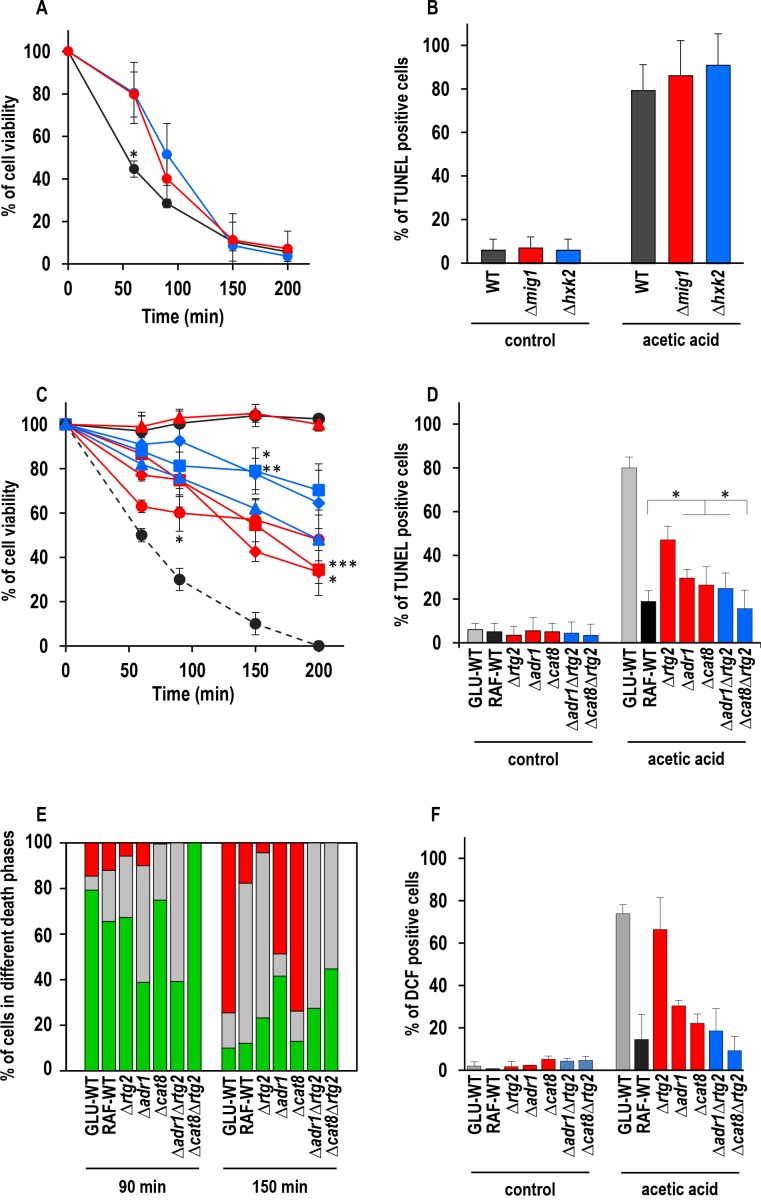
FIGURE 2: Effect of genetic inactivation of both RTG and CCR pathways on AA-PCD in either glucose- or raffinose-grown cells. **(A)** Wild-type (WT, black), Δ*mig1 *(red) and Δ*hxk2* (blue) mutant cells were treated with 80 mM acetic acid in growth medium with glucose as carbon source. Cell viability was analyzed by measuring colony-forming units (cfu) at indicated times. Cell survival based on the cfu was set at 100% at 0 min. The means of five independent experiments with standard deviations are reported. Anova-Bonferroni test: statistically different with (*) p < 0.001 when comparing WT with Δ*mig1* or Δ*hxk2* mutant cells. **(B)** DNA fragmentation in cells grown in glucose was detected by the TUNEL assay using confocal microscopy analysis. Percentage of TUNEL-positive cells is reported at 150 min. At least 400 cells were analyzed in three samples from each of three independent experiments. WT, grey bars; Δ*mig1, *red bars; Δ*hxk2*, blue bars. **(C)** GLU-WT (●, dashed line) and RAF-WT (●, black line), Δ*rtg2* (●, red line), Δ*adr1* (■, red line), Δ*cat8* (♦, red line), Δ*hap4 *(▲, red line), Δ*adr1Δrtg2* (■, blue line), Δ*cat8*Δ*rtg2* (♦, blue line) and Δ*hap4*Δrtg2 (▲, blue line) cells were treated with acetic acid either in glucose or in raffinose as carbon source. Cell viability was analyzed at indicated times by measuring colony-forming units (cfu). Cell survival (100%) corresponds to the cfu at time zero. The means of five independent experiments with standard deviations are reported. Anova-Bonferroni test: statistically different with (*) p < 0.01 when comparing Δ*rtg2* versus Δ*cat8*Δ*rtg2* at 90 min, Δ*adr1* with Δ*adr1*Δ*rtg2* at 150 min or both Δ*adr1* and Δ*cat8 *with Δ*cat8*Δrtg2 at 200 min; (**) p < 0.001 when comparing Δ*cat8* with Δ*cat8*Δ*rtg2* at 150 min; (***) p < 0.0001 when comparing Δ*adr1* with Δ*adr1*Δ*rtg2* at 200 min. **(D)** DNA fragmentation was detected by TUNEL assay using confocal microscopy analysis. Percentage of TUNEL-positive cells is reported at 150 min. At least 400 cells were analyzed in three samples from each of three independent experiments. Fisher’s exact test: statistically different with (*) p < 0.01 when comparing Δ*adr1*, Δ*cat8* or Δ*adr1*Δ*rtg2* versus WT or Δ*cat8*. GLU-WT, grey bars; RAF-WT, black bars; single knock-out cells, red bars; double knock-out cells, blue bars. **(E) **In a typical experiment GLU-WT, RAF-WT and knock-out cells grown in raffinose as indicated, were treated with acetic acid (AA). The cells were collected at 90 and 150 min after AA treatment, co-stained with Annexin V-FITC/PI and analyzed by confocal microscopy to measure PS externalization on the cell surface. The bars indicate the percentage of stained cells at different stages of death: early apoptotic cells (Annexin V^+^/PI^-^, green); late apoptotic cells (Annexin V^+^/PI^+^, grey); necrotic cells (Annexin V^-^/PI^+^, red). At least 400 cells were counted for each sample at indicated time. The experiment was repeated twice, with virtual identical results. Fisher’s exact test: statistically different with p < 0.005 when comparing Annexin V^+^ Δ*adr1* or Δ*adr1*Δ*rtg2* *versus* all other cell types at 90 min, when comparing PI^+^ Δ*adr1* versus Δ*cat8* at 150 min and when comparing Annexin V^+^ Δ*adr1* or Δ*cat8*Δ*rtg2* *versus* all other cell types at 150 min. **(F)** GLU-WT, RAF-WT and knock-out cells grown in raffinose, as indicated, were incubated in the absence (control) or in the presence of acetic acid for 30 min, then collected and stained with H_2_DCF-DA. DCF-stained cells due to ROS accumulation were analyzed by confocal microscopy. Bars indicate the percentage of DCF-positive cells with SD, calculated by counting at least 400 cells in three independent experiments. GLU-WT, grey bars; RAF-WT, black bars; single knock-out cells, red bars; double knock-out cells, blue bars.

Genes involved in CCR are of two types: genes such as *HXK2* and *MIG1*, which are active in glucose-grown cells and repress certain transcription factors such as Hap4 and Cat8; genes required for de-repression, such as *SNF1* and its downstream-activated transcription factors, Adr1 and Cat8*, *which are active in cells grown on alternative carbon sources, like raffinose (Fig. 1). In order to investigate the relations between glucose repression and the determination of cell fate, we first analyzed the effect of *MIG1* or *HXK2* deletions on AA-PCD sensitivity under glucose repression conditions. *MIG1* encodes a DNA-binding zinc-finger protein that forms a part of a central transcriptional repressor complex, exerting its function on many genes, including those encoding for respiratory, gluconeogenic and alternative carbon source utilization proteins, whereas *HXK2* encodes for hexokinase 2, the principal glucose phosphorylation enzyme and a putative glucose sensor 4. Δ*mig1 *and Δ*hxk2 *mutant cells were treated with 80 mM acetic acid in a medium containing 2% glucose and cell survival was analyzed over time and compared to that of wild-type (WT) cells (Fig. 2A). Both Δ*mig1* and Δ*hxk2* mutant cells progressively lost their viability within 200 min, when more than 90% of the cells were unviable. Comparison with GLU-WT cells revealed a higher percentage of survival at 60 min (about 80% for Δ*mig1 *and Δ*hxk2* mutants versus ~45% for WT), indicating a transient delay in cell death in these two mutants in response to acetic acid treatment. DNA fragmentation was analyzed in these cells to evaluate the nature of cell death. At 150 min, about 90% of Δ*mig1 *or Δ*hxk2* cells were positive in the TUNEL assay (Fig. 2B), similar to TUNEL-positive GLU-WT cells (80%). In the absence of acetic-acid treatment, less than 5% of all three strains analyzed were TUNEL-positive. These data show that perturbations in glucose repression by deleting *MIG1* or *HXK2* did not affect final AA-PCD in glucose-grown cells. Then, we analyzed Δ*mig1*Δ*mks1 *and Δ*hxk2*Δ*mks1 *cells to study the effect of simultaneous constitutive activation of the RTG pathway and CCR inactivation on AA-PCD in glucose. We found that Δ*mig1*Δ*mks1 *cells completely lost viability like WT and single knock-out cells, whereas Δ*hxk2*Δ*mks1* showed 20% survival after 200 min acetic acid treatment (Fig. 1S).

### *ADR1* and *CAT8* cooperate with *RTG2* to induce evasion of yeast AA-PCD in raffinose

In contrast to glucose-grown cells, raffinose-grown cells, in which mitochondrial respiration is de-repressed, evade AA-PCD 16. *HAP4, ADR1* and *CAT8* encode transcriptional activators that control expression of glucose-repressible genes 30. They are directly or indirectly activated by *SNF1*, the yeast homologue of AMP-activated protein kinase (AMPK), only under glucose depletion or with alternative carbon sources, when metabolism is shifted to aerobic respiration. Since *SNF1* is an essential gene for cell growth in raffinose in W303-1B background strains, to investigate the role of glucose-repressible transcriptional factors in evasion of yeast AA-PCD under glucose de-repression conditions, viability of AA-treated Δ*hap4*, Δ*adr1* or Δ*cat8 *mutant cells was analyzed in raffinose as a function of time together with GLU-WT and RAF-WT cells, which undergo and evade AA-PCD, respectively, as controls (Fig. 2C). Δ*rtg2 *mutant cells, which undergo AA-PCD due to inactivation of the RTG pathway 16, were also analyzed. Δ*adr1* and Δ*cat8 *mutant cells progressively lost their viability reaching about 35% after 200 min, whereas, as expected, GLU-WT cells completely lost their viability, RAF-WT remained 100% viable and Δ*rtg*2 cell viability decreased to about 50% at the same time point (Fig. 2C). Δ*hap4 *cells were as viable as RAF-WT after 200 min, consistent with our published results 16. Accordingly, the percentage of cells showing DNA fragmentation, as judged by TUNEL assay, was inversely proportional to cell viability at 150 min for all cell types analyzed (RAF-WT, 20%; Δ*adr1*, 30%; Δ*cat8,* 25%; GLU-WT, 80%) except in the case of Δ*rtg*2 (47%) (Fig. 2D). Thus, *RTG2*, *CAT8* and *ADR1* are all important for cells to evade AA-PCD in raffinose as the carbon source.

In order to investigate potential interactions between *RTG2 *and *CAT8* or *ADR1* in determining yeast cell evasion of AA-PCD, we generated Δ*adr1*Δ*rtg2* and Δ*cat8*Δ*rtg2* double mutant strains and determined cell survival *en route* to AA-PCD. Δ*hap4*Δ*rtg2* cells 16 were also analyzed. Surprisingly, cell viability measured during AA treatment was significantly higher for Δ*adr1*Δ*rtg2* and Δ*cat8*Δ*rtg2* double mutants compared to the respective single mutants, with ~79% and 70% viability at 150 and 200 min, respectively, for the double mutants versus about 50% to 34% for the single mutants (Fig. 2C). The same behaviour was shown by Δ*adr1*Δ*rtg3* and Δ*cat8*Δ*rtg3 *cells lacking the Rtg2-dependent transcriptional complex Rtg1/3 (Fig. 1S). Δ*hap4*Δ*rtg2* cells lost viability essentially as with the Δ*rtg2* single mutant. However, whereas the Δ*cat8*Δ*rtg2* double mutant showed a small but significant reduction in DNA fragmentation as compared with the Δ*cat8 *and Δ*rtg2 *single mutants, the percentage of Δ*adr1*Δ*rtg2* TUNEL-positive cells was similar to that of Δ*adr1 cells* (Fig. 2D). As expected, in the absence of AA-treatment, less than 5% of the cells were TUNEL-positive.

Since double knock-out cells showed virtually the same percentage of DNA fragmentation as single knock-out cells, although with a higher viability, we looked more closely at the nature of the death process in WT and knock-out cells and measured PS externalization on the cell surface and reactive oxygen species (ROS) accumulation. We first analyzed WT and knock-out cells *en route* to AA-induced cell death by Annexin V/PI co-staining and confocal microscopy as a function of time. PI staining of cells indicates loss of plasma membrane integrity. By Annexin V/PI co-staining it is possible to discriminate among early-apoptotic (Annexin V^+^/PI^-^), late apoptotic/secondary necrotic (Annexin V^+^/PI^+^) or necrotic (PI^+^) cells (Fig. 2E) 31. Semiquantitative analysis of the percentage of stained cells at different stages of death was performed (Fig. 2E). All cell types were analyzed before AA-treatment as controls. Virtually no PI and < 5% Annexin V stained cells were detected.

For GLU-WT cells, notwithstanding the high percentage of cells in the early-apoptotic stage at 90 min (80%), the percentage of necrotic and late-apoptotic cells was 75% and 15%, respectively, at 150 min, when cell viability is virtually lost (see Fig. 2A). In the case of RAF-WT cells, which evaded AA-PCD but did not grow, a percentage of early-apoptotic cells was still detectable at 90 min (65%), while the number of necrotic cells decreased to 15% and that of late-apoptotic cells were 70% at 150 min, when cell viability is 100%, in fairly good agreement with the number of cells with DNA fragmentation (see Fig. 2D). The percentage of Δ*rtg2* cells in raffinose at different stages of death was found to be similar to that of RAF-WT cells with almost no necrotic cells, whereas *CAT8* or *ADR1* deletion resulted in the virtual restoration of the necrotic phenotype of dead GLU-WT cells at 150 min, when all three knock-out cells showed 40-50% viability. Interestingly enough, *RTG2* deletion in either Δ*cat8 *or Δ*adr1* cells caused only apoptosis-like behaviour and no necrotic cell death at 150 min with about 70% cell viability. Then, since AA-PCD cells have been shown to accumulate ROS early *en route *to death, we also measured ROS after 30 min of acetic-acid treatment (Fig. 2F) 32. GLU-WT cells showed the highest level (66%) of ROS, with 15% in RAF-WT cells and 55% in Δ*rtg2* cells in raffinose showing oxidative stress, in fairly good agreement with 16. Δ*adr1* and Δ*cat8 *cells, which lost viability similarly to Δ*rtg2 *cells but with more necrotic cells, showed a lower level of ROS accumulation at 30 min (30% and 22%, respectively) (cf Fig. 2E and F). Δ*adr1*Δ*rt*g2 and Δ*cat8*Δ*rtg2 *cells, which behaved more like RAF-WT cells (with no necrotic cells) showed the lowest ROS level (18% and 9%, respectively).

Taken together, these data show that similar to *RTG2*, *ADR1* and *CAT8 *also contribute to AA-PCD evasion under glucose de-repressing conditions, even though *RTG2* deletion showed a different effect in WT or Δ*adr1*/Δ*cat8 *cells. Partial recovery of the ability of cells to evade AA-PCD in Δ*cat8*Δ*rtg2* and Δ*adr1*Δ*rtg2* double mutant cells showed unexpectedly that *RTG2* is a suppressor of either *CAT8 *or *ADR1* deletion. To get an insight into the mechanism of *RTG2* suppression, we prepared Δ*adr1*Δ*cat8*Δ*rtg2*, Δ*cat8*Δ*mks1* and Δ*adr1*Δ*mks1* cells and analyzed their viability after 200 min acetic acid treatment. Either the triple knock-out cells or the double knock-out cells, with constitutive activation of the RTG pathway, restored a WT phenotype (97%, 87% and 81% viability, respectively, at 200 min) like the double knock-out cells with the RTG pathway inactivated (Fig. 1S). Altogether these data suggest the Adr1 and Cat8 interact with Rtg2 and with each other in inducing cell resistance to AA-PCD in raffinose. In the absence of Adr1 and Cat8, AA-PCD evasion is acquired through activation of an alternative factor/pathway repressed by RTG2.

### RTG pathway activation depends on *ADR1* and *CAT8* in raffinose-grown cells

**Figure 3:**
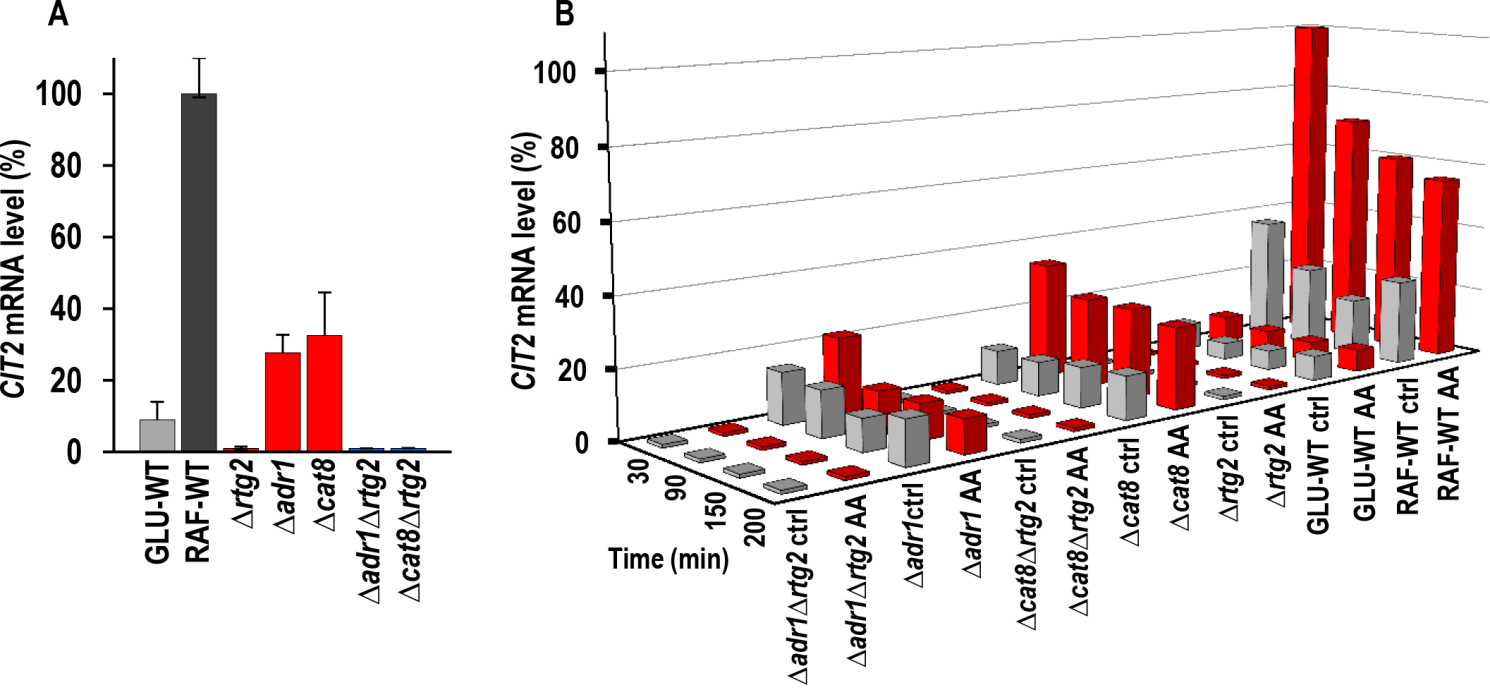
FIGURE 3: *CIT2 *mRNA level in raffinose-grown wild-type and mutant strains in exponential phase growth and *en route* to acetic acid treatment. **(A) ***CIT2* mRNA levels were measured by real-time PCR in RAF-WT cells and in deletion mutant cells grown in raffinose medium and collected in exponential phase (OD_600_ 0.6-0.7). GLU-WT cells were also analyzed as control. Percentage of *CIT2* mRNA levels, normalized to *ACT1* mRNA, as compared with that in RAF-WT cells (100%), was reported. **(B)** *CIT2 *mRNA levels were measured by real-time PCR at the indicated time points in wild-type and mutant strains grown in raffinose medium with (AA, red bars) or without (ctrl, grey bars) the supplementation of 80 mM acetic acid**. **GLU-WT samples were included as controls. Percentage of *CIT2* mRNA levels, normalized to *ACT1* mRNA, as compared with that in AA-treated RAF-WT cells at 30 min, was reported.

Since it has previously been shown that RAF-WT evades AA-PCD by activating the RTG pathway, to gain insights into interaction between *ADR1/CAT8* and the RTG pathway we analyzed mRNA expression of *CIT2*, whose up-regulation is a marker of RTG pathway activation 17, in Δ*rtg2*, Δ*adr1*, Δ*cat8*, Δ*adr1*Δ*rtg2*, Δ*cat8*Δ*rtg2* mutant strains along with WT controls. As a preliminary, *CIT2* mRNA level was measured in mutant strains in exponential growth in raffinose as well as in GLU- and RAF-WT cells, as controls (Fig. 3A). The *CIT2* mRNA level in GLU-WT cells was found to be ~13-fold lower than that in RAF-WT cells, which was taken as 100%, as previously found 18. Both *ADR1* and *CAT8* deletion caused a ~4-fold reduction in the *CIT2* mRNA level in raffinose, as compared with RAF-WT. A complete abolishment of *CIT2 *expression was found in Δ*rtg2,* Δ*adr1*Δ*rtg2 *and Δ*cat8*Δ*rtg2* mutant cells as expected. We then analyzed activation of RTG pathway over time in raffinose-grown WT and mutant cells with or without acetic acid treatment (Fig. 3B). GLU-WT cells *en route* to AA-PCD were also analyzed for comparison. A 3-4-fold *CIT2* up-regulation was found in acetic acid-treated RAF-WT cells with respect to non-treated control samples at all time points analyzed, indicating RTG pathway activation in fairly good agreement with our published result 16. In Δ*adr1 *mutant cells, acetic acid treatment had little effect on *CIT2 *mRNA levels over the 200-min period (compare Δ*adr1 *ctrl with Δ*adr1 *AA in Fig. 3B). Importantly, *CIT2 *mRNA levels were lower in Δ*adr1* mutant cells than in acetic acid-treated, RAF-WT cells (compare Δ*adr1* AA with RAF-WT AA in Fig. 3B), suggesting that Δ*adr1 *reduces cell ability to evade AA-PCD in raffinose medium possibly by limiting the activation of the RTG pathway. Similarly, no activation of the RTG pathway occurred in GLU-WT cells *en route* to AA-PCD, as previously reported 16.

Interestingly, in Δ*cat8 *cells that undergo AA-PCD to a similar extent as in Δ*adr1* cells*,* *CIT2 *mRNA levels were 2-3-fold higher in acetic acid-treated samples compared to untreated ones at all time points (compare Δ*cat8* AA with Δ*cat8* control in Fig. 3B), although the increase was at a lower degree than what was observed in RAF-WT cells. *CIT2* expression was completely abolished in Δ*rtg2,* Δ*adr1 *Δ*rtg2 *and Δ*cat8*Δ*rtg2 *cells either during AA treatment or in controls over the 200-min treatment period. The expression of *DLD3*, another RTG-target gene 33, was also measured. During exponential cell growth, differently from *CIT2*, *DLD3* mRNA level in GLU-WT cells was 2-fold higher than in RAF-WT cells, but its expression, although strictly requiring *RTG2*, is neither regulated by *ADR1* or *CAT8 *(Fig. 2SA) nor by acetic acid treatment in de-repression conditions (Fig. 2SB). Notwithstanding RTG pathway inactivation, AA-PCD evasion is observed in Δ*adr1*Δ*rtg2 *and Δ*cat8*Δ*rtg2* double mutants differently from Δ*rtg2* or Δ*adr1 *and Δ*cat8 *single mutants, in which a low *CIT2* expression was detected (Figure 3B). This suggests that activation of the RTG pathway is not the sole factor leading to AA-PCD resistance in RAF-WT cells. Rather, Δ*adr1*Δ*rtg2 *and Δ*cat8*Δ*rtg2 *double mutant strains acquired AA-PCD resistance through rewiring of other pathways.

## DISCUSSION 

Mitochondrial retrograde signaling has been shown to be dependent on the carbon source 34 and to cross-talk with other signaling pathways, including the CCR pathway, in response to mitochondrial dysfunction 22. In this study we analyzed the relations between these two signaling pathways in the cell fate decision of yeast cells in response to acetic acid stress. We found that Adr1 and Cat8*, *two transcription factors dependent on Snf1, which regulates physiological reprogramming dependent on carbon-source availability, are strictly required for the activation of *RTG2*-dependent transcription in respiratory de-repression conditions, both during exponential growth, as judged by the strong *CIT2* mRNA down-regulation found in both single mutants (Fig. 3A), and in AA-treated cells, in which deletion of either transcription factor causes increased cell death and down-regulation of *CIT2* expression with respect to RAF-WT cells, which evade AA-PCD (cf Figs. 2C, D and 3B). Thus, in RAF-WT cells AA-PCD evasion is directly dependent on mitochondrial RTG pathway activation, as judged by the expression of *CIT2*, the prototypical RTG-target gene.

**Figure 4:**
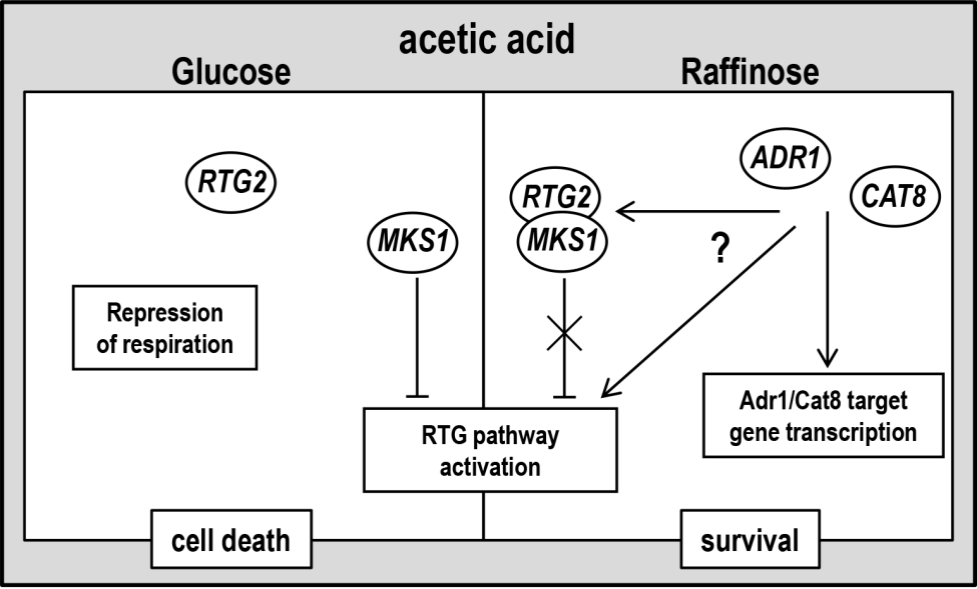
FIGURE 4: A working hypothesis model for yeast acetic acid-stress response regulation by RTG and CCR pathways. Exponential glucose-grown cells undergo PCD in response to acetic acid with simultaneous repression of respiration and of RTG pathway. Raffinose-grown cells evade AA-PCD with de-repressed respiration 16 and RTG pathway activation. *ADR1* and *CAT8* are required for RTG pathway activation through an unknown mechanism (for details see text), showing a key role of *SNF1*-dependent CCR pathway in carbon source-regulation of mitochondrial RTG pathway. *RTG2* may play a function in promoting cell death in repressing conditions when RTG pathway is inactive.

The observed difference between the expression of two *RTG2*-regulated genes, *CIT2* and *DLD3*, shows how RTG-dependent mitochondrial retrograde signaling and the ensuing set of transcriptionally reprogrammed genes is strain-dependent. Indeed, *DLD3* expression was not induced in *ρ*^0^ cells of the same yeast strain used in this study 33. These data suggest that both *ADR1* and *CAT8* have a role in the determination of the up-regulated RTG-target genes and that Cat8/Adr1 and Rtg2 may regulate *CIT2* expression independently. Here we found that in cells lacking either Adr1 or Cat8, both *RTG2* and *RTG3 *deletion caused the same phenotype, i.e. restoration of AA-PCD evasion similar to RAF-WT cells (Figs. 2C and 1S). This, together with the observation that Δ*rtg2* and Δ*rtg3 *cells undergo AA-PCD in a similar way, as shown here and in 16, shows that Cat8/Adr1 may regulate RTG-target gene expression directly or indirectly (Fig. 4).

In this respect, our results shed new light on the physiological role of RTG pathway activation in the evasion on AA-induced cell death. In fact, the RTG-pathway seems to have different functions in WT and in Δ*adr1* or Δ*cat8 cells *in de-repression conditions. Although loss of viability in Δ*adr1* and Δ*cat8* cells was comparable to that observed with *RTG2-*lacking cells (Fig. 2A, B and 16), the effects of *RTG2* or *ADR1*/*CAT8* deletion on the nature of the cell death process were different, as judged by the analysis of some morphological and biochemical features of AA-induced cell death. Acetic acid appeared to induce both an apoptosis-like PCD and a necrotic death process in GLU-WT cells. In raffinose de-repressing conditions, activation of the RTG pathway induced evasion of cell death in response to acetic acid, but with a strong decrease only in necrotic cell death. *RTG2 *deletion virtually abolished the necrotic phenotype while *ADR1/CAT8* deletion decreased apoptosis-like PCD and increased necrosis. Moreover, in cells with functional *ADR1* and *CAT8* ROS level seemed to be highly dependent on RTG pathway activation, whereas in Δ*adr1* and Δ*cat8* cells, which seemingly have a lower mitochondrial activity, the ROS level was always significantly decreased and apparently independent on the RTG pathway (cf Figs. 2E, F and 3B). Altogether these data show that in respiratory-de-repressed raffinose-grown WT cells RTG pathway activation triggers an anti-oxidant, pro-survival cell response to acetic acid stress decreasing necrotic cell death. On the other hand, when the *SNF1-*axis of CCR pathway is inactivated by *ADR1* or *CAT8* deletion, RTG-dependent signaling causes a metabolic reprogramming leading to cell death. Thus genetic inactivation of the RTG pathway in Δ*adr1 *or Δ*cat8* cells can restore AA-PCD resistance. Since it has been shown that Rtg2 controls RTG signaling by reversibly binding the negative regulator, Mks1 35, it is tempting to speculate that Rtg2 may play a so far uncharacterized function in promoting (or repressing evasion from) necrotic cell death in glucose repressing condition, when RTG pathway is off, e.g. by dynamic interaction with an unknown factor/pathway.

Then, differently from in WT cells, AA-PCD evasion cannot be attributed to RTG pathway activation in the double mutants *Δadr1Δrtg2* or *Δcat8Δrtg2*, since *CIT2* expression is completely abolished in these cells, independent of acetic acid treatment (Figs. 2C, D and 3B). Thus the protective effect of raffinose on yeast AA-PCD likely requires also a *SNF1*-independent signaling pathway sensitive to carbon source or mitochondrial function. Since *CAT8* and *ADR1* co-regulate several glucose-sensitive genes and can act independently or synergistically 28,36,37, it could also be hypothesized that they can substitute for each other in the metabolic reprogramming of *Δadr1Δrtg2* and *Δcat8Δrtg2* cells in evading AA-PCD. This was excluded by our results since the triple knock-out cells *Δadr1Δcat8Δrtg2* behave in the same way as the double knock-out cells, strongly suggesting the activation of alternative pathway/s inducing AA-PCD resistance.

We have previously shown that AA-PCD evasion occurs only in metabolic conditions characterized by concomitant activation of the RTG pathway and relief of CCR 16. This study confirms that this is the case as demonstrated by the lack of effect on cell survival and DNA fragmentation upon genetic inactivation of CCR by deletion of *HXK2* or *MIG1* in glucose-grown cells (Fig. 2A, B), in which the RTG pathway is not activated by AA-treatment (Fig. 2E, F). This is in agreement with our previous observations that shift in glucose concentration from 2% to 0.5% can induce evasion of AA-PCD only in Δ*mks1* cells, in which the RTG pathway is constitutively activated 16. Consistent with this, we found that Δ*mig1*Δ*mks1 *and Δ*hxk2*Δ*mks1 *did not acquire AA-PCD evasion in 2% glucose (Fig. 1S). This is because neither *HXK2 *nor *MIG1 *deletion phenocopies glucose de-repression, which occurs in RAF-WT or 0.5% glucose-grown cells. Moreover, the deletion of *HAP4*, whose transcription is under the negative control of the Mig1/Hxk2 complex, did not abolish AA-PCD evasion in raffinose-grown cells, suggesting that the Mig1/Hxk2 axis of CCR does not significantly contribute to glucose-dependent control of yeast acetic acid-stress sensitivity. It is of note that enhancement of AA-PCD by deletion of *HXK2* has been found in a different yeast strain 38. This could be due in part to the different degree of activation of CCR and the cAMP/PKA signaling pathway in different genetic background and growth conditions 39.

Thus, metabolic reprogramming caused by concomitant activation of the RTG pathway and *SNF1*-dependent relief of CCR appears to be required for AA-PCD evasion. Genome-wide transcriptome analyses have shown both that *RTG2*-dependent genes are involved in peroxisomal biogenesis and anaplerotic reactions of tricarboxylic acid cycle intermediates, directing carbon metabolism to α-ketoglutarate biosynthesis 40, and that *ADR1*-dependent genes channel metabolites into acetyl-CoA production 28. Yeast cells grown on glucose cannot metabolize acetic acid due to activation of CCR (41 and refs therein) explaining in part why yeast is sensitive to acetic acid stress in the presence of glucose. Yet, our data show that relief of CCR, without up-regulation of RTG-target genes, is not sufficient to induce AA-PCD evasion. Intracellular acetate is transformed into acetyl-CoA through *ACS1* and *ACS2*, which can be used as a metabolite for metabolic pathways or a substrate for lysine acetyltransferases in epigenetic regulation of gene expression through histone acetylation 42. In view of the role of *RTG2*, *ADR1* and *CAT8* in chromatin remodeling 36,43 it is tempting to speculate that simultaneous activation of the RTG pathway and *SNF1/AMPK*-target gene expression can signal metabolic control of AA-PCD through modulation of acetyl-CoA levels, as already shown for respiratory-deficient ageing yeast cells 44. Our data confirm the use of yeast as a model for a better understanding of cell adaptations to the extracellular environment and revealing the molecular basis of PCD evasion in eukaryotes.

## Materials and Methods

### Yeast strains, growth conditions and acetic acid treatment

The *S. cerevisiae* strains used in this study are listed in Table 1. Cells were grown at 30°C in YPD or YPR (1% yeast extract, 2% bactopeptone, and 2% glucose or raffinose, respectively). Acetic acid treatment was carried out as described 45. Briefly, cells were grown at 26°C up to exponential phase (OD_600_ = 0.6 - 0.8) in YPD or YPR, resuspended (10^7^ cells/ml) in the same medium adjusted to pH 3.00 with HCl, with or without the supplementation of 80 mM acetic acid and incubated for different times at 26°C. Cell viability was determined by measuring colony forming units (cfu) after 2 days of growth on YPD plates at 30°C.

**Table 1:** Strains of *Saccharomyces cerevisiae* used in this study.

**Strain (name)**	**Genotype**	**Reference/source**
W303-1B (WT)	*MATα ade2 leu2 his3 trp1 ura3*	
Δ*hxk2*	W303-1B *hxk2*Δ*::kanMX4*	This study
Δ*mig1*	W303-1B *mig1*Δ*::kanMX4*	This study
Δ*rtg2*	W303-1B *rtg2*Δ::LEU2	16
Δ*hap4*	W303-1B *hap4*Δ::*kanMX4*	16
Δ*adr1*	W303-1B *adr1*Δ*::kanMX4*	This study
Δ*cat8*	W303-1B *cat8*Δ*::kanMX4*	This study
Δ*hap4*Δ*rtg2*	W303-1B *hap4*Δ*::kanMX4 rtg2*Δ*::LEU2*	16
Δ*adr1*Δ*rtg2*	*W303-1B adr1*Δ*::kanMX4 rtg2*Δ*::LEU2*	This study
Δ*cat8*Δ*rtg2*	W303-1B *cat8*Δ*::kanMX4 rtg2*Δ*::LEU2*	This study
Δ*adr1*Δ*rtg3*	W303-1B *adr1*Δ*::kanMX4 rtg3*Δ*::LEU2*	This study
Δ*cat8*Δ*rtg3*	W303-1B *cat8*Δ*::kanMX4 rtg3*Δ*::LEU2*	This study
Δ*adr1*Δ*mks1*	W303-1B *adr1*Δ*::kanMX4 mks1*Δ*::LEU2*	This study
Δ*cat8*Δ*mks1*	W303-1B *cat8*Δ*::kanMX4 mks1*Δ*::LEU2*	This study
Δ*mig1*Δ*mks1*	W303-1B *mig1*Δ*::kanMX4 mks1*Δ*::LEU2*	This study
Δ*hxk2*Δ*mks1*	W303-1B *hxk2*Δ*::kanMX4 mks1*Δ*::LEU2*	This study
Δ*adr1*Δ*cat8*Δ*rtg2*	W303-1B *adr1*Δ*::kanMX4 cat8*Δ*::HIS3 rtg2*Δ*::LEU2*	This study

### Mutant strain construction

To delete *ADR1*, *CAT8*, *HXK2*, and *MIG1* genes in W303-1B, disruption cassettes with the *kanMX4* selection marker were amplified by PCR using genomic DNA from respective deletion mutant strains in the BY4741 strain background (Yeast genome deletion project) as template. PCR products of the disruption cassettes were transformed into W303-1B strain using the high efficiency yeast transformation method 46. Gene disruption was confirmed by PCR genotyping. To generate Δ*adr1*Δ*rtg2* and Δ*cat8*Δ*rtg2* double mutant strains, a plasmid carrying an *rtg2::LEU2* disruption cassette (pUCrtg2::*LEU*2, 47) was digested with *Pst*I and transformed into Δ*adr1* or Δ*cat8* single knock-out strains. To generate Δ*adr1*Δ*rtg3* and Δ*cat8 *Δ*rtg3* strains, a Δ*rtg3::LEU2 *cassette was amplified from Δ*rtg3* cells 16 using the primer pair (F) 5’- CGAAAGTGAGGCTGAGAACC-3’ and (R) 5’-GACTCTCCATAGTG-CCAGCA-3’ and transformed into Δ*adr1* or Δ*cat8* single knock-out strains. Δ*rtg2 *and Δ*rtg3 *mutations were confirmed by the glutamate auxotrophy phenotype and PCR genotyping. For generating the Δ*cat8*Δ*adr1*Δ*rtg2 *strain, we first cloned pBS-Δ*cat8*::kanmX4 and then replaced the kanMX4 cassette with a *HIS3 *cassette. pBS-cat8::HIS3 was used to transform Δ*adr1*Δ*rtg2* knock-out cells. To generate Δ*adr1*Δ*mks1*, Δ*cat8*Δ*mks1*, Δ*mig1*Δ*mks1* and Δ*hxk2*Δ*mks1* double mutants, a Δ*mks1*::LEU2 disruption cassette was introduced into respective single mutant strains and transformants were selected on minimal medium lacking leucine. Δ*mks1*::LEU2 mutations were confirmed by PCR-genotyping.

### TUNEL assay, Annexin V/PI staining and ROS detection

DNA fragmentation was detected by TUNEL assay as reported in 16. Briefly, acetic acid-treated and untreated control cells (2 x 10^7^) were harvested at the 150-min time point, fixed in 3.7% formaldehyde solution in PBS, digested with 750 μg/ml zymolyase 20T and incubated in permeabilization solution (0.1% Triton-X100, 0.1% sodium citrate) for 2 min on ice, and then 30 μl TUNEL reaction mixture was added (In Situ Cell Death Detection kit, Fluorescein, Roche) for 1 hour at 37°C. After incubation cells were washed, resuspended in PBS and observed using a Leica TCS SP5 confocal microscope. Plasma membrane integrity was measured by propidium iodide (PI) staining essentially as described in 48. Briefly, 1 x 10^7 ^cells were centrifuged at 10,000 g for 3 min and resuspended in 10 μl medium, treated with 0.5 μl PI (500 μg/ml), incubated for 15 min at room temperature in the dark and observed with a confocal microscope (see below). PS exposure on the cell surface and membrane integrity were detected by a fluorescein isothiocyanate (FITC)-coupled Annexin V reaction, using the Annexin-V-FLUOS Staining Kit (Roche). AA-treated and control yeast cells (2 x 10^7^) were sedimented at 10,000 g for 3 min at different times and digested with 750 μg/ml zymolyase 20T in sorbitol buffer (1.2 M sorbitol, 0.5 mM MgCl_2_, 35 mM potassium phosphate pH 6.8) at 30°C for 1 h. Cells were washed twice with binding buffer (10 mM HEPES/NaOH pH 7.4, 140 mM NaCl, 2.5 mM CaCl_2_, 1.2 M sorbitol) and 30 μl label solution (2 μl Annexin V, 2 μl PI, 98 μl binding buffer) were added to 50 μl cell suspension (1 x 10^7^) for 20 min incubation in the dark at room temperature. After washing, the cells were applied to microscopic slides and observed using LEICA TCS SP5 confocal microscopy (HCX PL APO lambda blue 63 x 1.40 objective) exciting the sample with a Argon Laser at 488 nm and emission at 494 - 537 nm, for FITC-Annexin V, and at 629 - 776 nm, for PI. Digital images were analyzed using LAS X software. To detect intracellular ROS levels, 10 μg/ml 2’,7-dichlorofluorescein diacetate (H_2_DCF-DA; Molecular Probes) dissolved in ethanol was added to cells both 30 min before and during cell treatment with or without AA. AA-treated or control cells were harvested at different times and oxidation to the fluorophore dichlorofluorescein (DCF) was detected by confocal fluorescence microscopy analysis as above, with excitation at 488 nm and emission at 500-600 nm.

### Real-time polymerase chain reaction (PCR)

*CIT2* and *DLD3* mRNA levels were determined in acetic acid-treated or control cells collected at the exponential phase (OD_600_ = 0.7). 20 ml of cell cultures were withdrawn at different times and centrifuged at 3000 g. Cells were either stored at −80°C or immediately lysed with by zymolyase 20T to extract total RNA using Presto™ Mini RNA Yeast Kit (Geneaid Biotech Ltd). 1 μg RNA (OD_260_/OD_280_ ≥ 1.9) was immediately reverse-transcribed using QuantiTect® Reverse Transcription Kit (Qiagen) and cDNA used for real-time PCR analysis or stored at −20°C. Real-time PCR was carried out using a QuantiTect® SYBR Green PCR Kit (Qiagen) on an Applied Biosystems QuantStudio™ 6 Flex machine using the following primer pairs: for *CIT2*: (F) 5′-CGGTTATGGTCATGCTGTGCT-3′ and (R) 5′- GGTCCATGGCAAACTTACGCT-3′; for *ACT1*: (F) 5′-CTTTGGCT-CCATCTTCCATG-3′ and (R) 5′-CACCAATCCAGACGGAGTACTT-3′; for *DLD3* see 49. The fold-increase (2^-ΔΔCt^) of *CIT2* and *DLD3 *mRNA levels, normalized to *ACT1* mRNA, as compared with that in RAF-WT cells was calculated and reported as the percentage of RAF-WT *CIT2* mRNA level taken as 100%.

## SUPPLEMENTAL MATERIAL

Click here for supplemental data file.

All supplemental data for this article are also available online at http://microbialcell.com/researcharticles/the-transcription-factors-adr1-or-cat8-are-required-for-rtg-pathway-activation-and-evasion-from-yeast-acetic-acid-induced-programmed-cell-death-in-raffinose/.
