# Supplementary material for: The transcription factors ADR1 or CAT8 are required for RTG pathway activation and evasion from yeast acetic acid-induced programmed cell death in raffinose
Source: Microb Cell. 2016 Dec 2;3(12):621–31. doi: 10.15698/mic2016.12.549 (PMC5348981; doi:10.15698/mic2016.12.549)
Supplement: Supplementary file 1 [file mic-03-621-s01.pdf]

Supplementary data to the manuscript entitled "The transcription factors ADR1 or CAT8 are required for RTG pathway activation and evasion from yeast acetic acid-induced programmed cell death in raffinose".

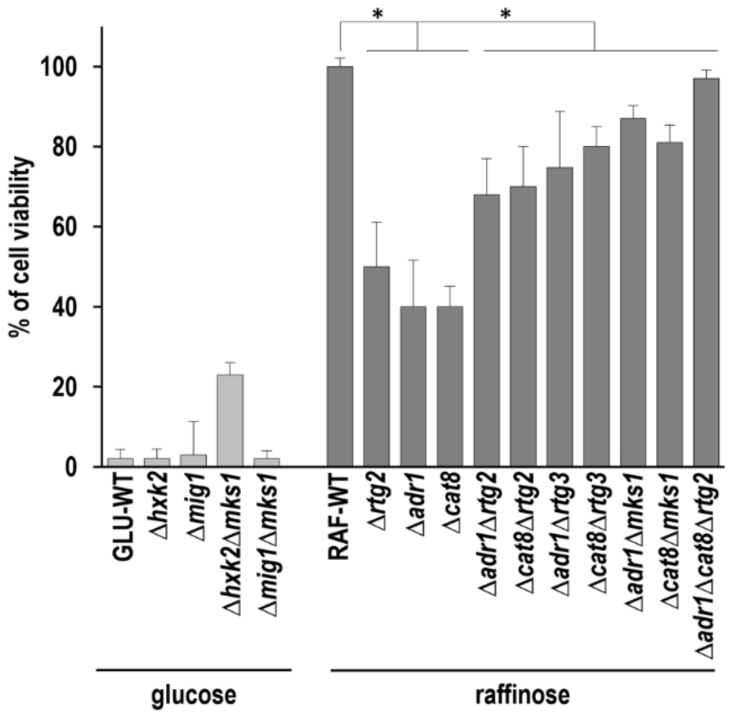

Figure 1S. Acetic acid sensitivity in glucose- and raffinose-grown wild-type and mutant cells. GLU-WT, RAF-WT and knock-out cells as indicated were treated with 80 mM acetic acid in glucose (light grey bars) or raffinose (dark grey bars) as carbon source. Cell viability was analyzed by counting colony-forming units (cfu) at 200 min. Cell survival based on the cfu was set at 100% at 0 min. The means of three independent experiments with standard deviations are reported. Anova-Bonferroni test: statistically different with (*) $p < 0.01$ when comparing Δ*adr1* or Δ*rtg2* or Δ*cat8* *versus* all other cell types.

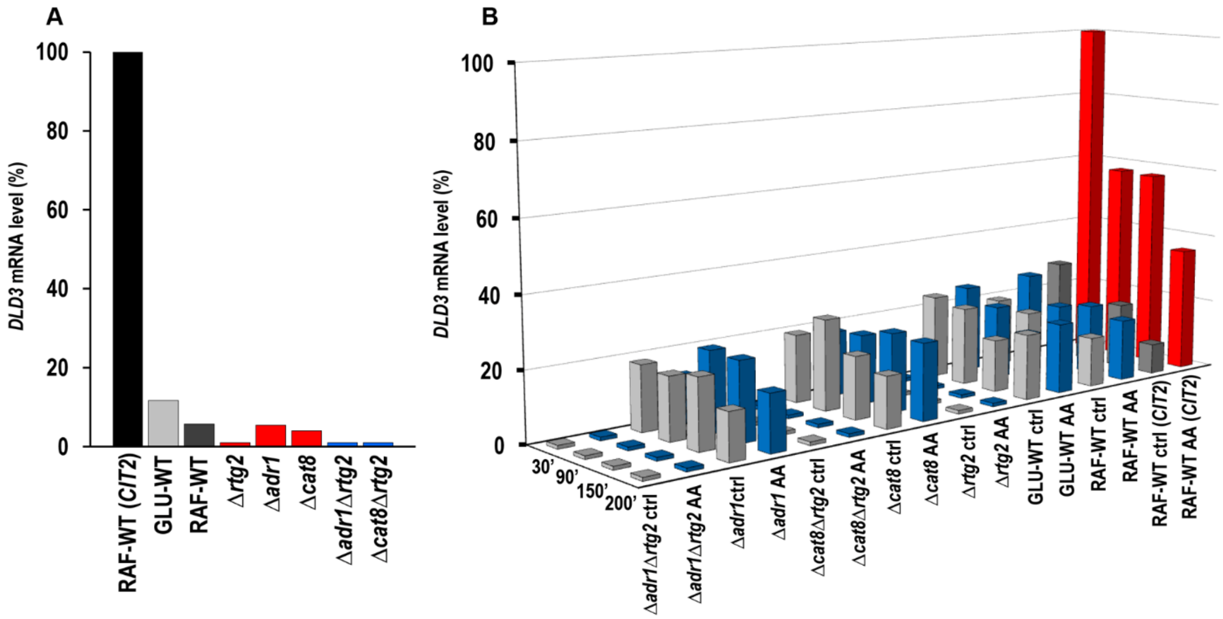

Figure 2S. *DLD3* mRNA level in raffinose-grown wild-type and mutant strains in exponential phase growth and *en route* to acetic acid treatment. (A) *DLD3* and *CIT2* mRNA levels were measured by real-time PCR in GLU-WT (grey bar) and RAF-WT (black bar) cells and in single (red bars) and double (blue bars) deletion mutant cells grown in raffinose medium and collected in exponential phase (OD_{600} 0.6-0.7). Percentage of *DLD3* mRNA levels, normalized to *ACT1* mRNA, as compared with the mRNA level of *CIT2* in RAF-WT cells (set to 100%, see fig. 5), was reported. (B) *DLD3* mRNA levels were measured by real-time PCR at the indicated time points in RAF-WT and mutant strains grown in raffinose with (AA, blue bars) or without (ctrl, grey bars) 80 mM acetic acid. GLU-WT cells were also analyzed as control. *CIT2* mRNA was also analyzed in RAF-WT (dark grey and red bars) as controls. Percentage of *DLD3* mRNA levels, normalized to *ACT1* mRNA, as compared with that mRNA level of *CIT2* in AA-treated RAF-WT cells at 30 min (set to 100%, see fig. 6), was reported.
